# Containment, Contact Tracing and Asymptomatic Transmission of Novel Coronavirus Disease (COVID-19): A Modelling Study

**DOI:** 10.3390/jcm9103125

**Published:** 2020-09-27

**Authors:** Ryo Kinoshita, Asami Anzai, Sung-mok Jung, Natalie M. Linton, Takeshi Miyama, Tetsuro Kobayashi, Katsuma Hayashi, Ayako Suzuki, Yichi Yang, Andrei R. Akhmetzhanov, Hiroshi Nishiura

**Affiliations:** 1 Kyoto University School of Public Health, Yoshida-Konoe-cho, Sakyo-ku, Kyoto 606-8501, Japan; kinoshita.ryo.5a@kyoto-u.ac.jp (R.K.); asami.a.1333@gmail.com (A.A.); seductmd@med.hokudai.ac.jp (S.-m.J.); nlinton@gmail.com (N.M.L.); tootsieroll2910@gmail.com (T.K.); katsuma5miffy@gmail.com (K.H.); akmskorokoro@gmail.com (A.S.); 2 Graduate School of Medicine, Hokkaido University, Kita 15 Jo Nishi 7 Chome, Kita-ku, Sapporo-shi, Hokkaido 060-8638, Japan; luke_yang1993@yahoo.co.jp (Y.Y.); akhmetzhanov@gmail.com (A.R.A.); 3 Osaka Institute of Public Health, Nakamichi 1-3-69, Higashinari, Osaka 537-0025, Japan; takeshi.j.miyama@gmail.com; 4 CREST, Japan Science and Technology Agency, Honcho 4-1-8, Kawaguchi, Saitama 332-0012, Japan

**Keywords:** epidemiology, containment, asymptomatic, emerging infectious diseases

## Abstract

When a novel infectious disease emerges, enhanced contact tracing and isolation are implemented to prevent a major epidemic, and indeed, they have been successful for the control of severe acute respiratory syndrome (SARS) and Middle East respiratory syndrome (MERS), which have been greatly reduced without causing a global pandemic. Considering that asymptomatic and pre-symptomatic infections are substantial for the novel coronavirus disease (COVID-19), the feasibility of preventing the major epidemic has been questioned. Using a two-type branching process model, the present study assesses the feasibility of containing COVID-19 by computing the probability of a major epidemic. We show that if there is a substantial number of asymptomatic transmissions, cutting chains of transmission by means of contact tracing and case isolation would be very challenging without additional interventions, and in particular, untraced cases contribute to lowering the feasibility of containment. Even if isolation of symptomatic cases is conducted swiftly after symptom onset, only secondary transmissions after the symptom onset can be prevented.

## 1. Introduction

Since a major epidemic of the novel coronavirus disease (COVID-19) took off in Wuhan, Hubei province, China, the magnitude of the epidemic quickly sparked globally, leading us to observe a substantial number of cases across borders. By late January 2020, multiple local transmissions have been observed outside of China, bringing the world to judge the feasibility of local containment during the first wave [1]. On 11 March 2020, the World Health Organization (WHO) characterized COVID-19 as a pandemic, as the epicenter expanded to the United States and several countries in Europe. As of 22 September 2020, the geographic spread of COVID-19 has continued to expand, reaching over 31 million confirmed cases and 962 thousand confirmed deaths worldwide. Moreover, community transmission (massive cases without a direct link to a confirmed case) has been a concern for nations failing to control the disease without effective control measures.

Several key characters of COVID-19 transmission have been described, allowing better understanding of complications to control this disease. They include the capability to shed the virus prior to symptom onset [2,3] and the substantial proportion of asymptomatic cases, which in current knowledge, ranges from 15–45% [4,5,6]. Severe acute respiratory syndrome (SARS), caused by a closely related coronavirus (SARS associated coronavirus), is known to have been mostly accompanied by clinical symptoms [7], and in particular, transmissions have happened after the date of illness onset, especially shortly before developing pneumonia [2,8]. Very few asymptomatic infections for SARS have been documented (i.e., the asymptomatic ratio is very small), and asymptomatic individuals have not been documented to contribute to a substantial number of secondary transmissions [9]. For this reason, contact tracing and containment were considered as feasible in controlling the disease [10]. Compared with SARS, published studies have suggested that there is a substantial number of pre-symptomatic transmissions, and moreover, asymptomatic infections are not uncommon, posing a pressing question over the feasibility of containment of COVID-19 [11]. An early case report from China has revealed pre-symptomatic transmission within a family cluster [12]. The viral load among asymptomatically infected individuals has been shown to be comparable to symptomatic individuals, implying that the transmission potential among asymptomatically infected individuals may be substantial [13]. Additionally, 80% of secondary cases may have been caused by a small fraction of infected individuals, indicating high individual variations in the number of secondary transmissions [14].

During the early course of the COVID-19 pandemic, the possibility of containment was discussed by a mathematical model, accounting for pre-symptomatic transmission and asymptomatic infection, even when strong evidence of substantial asymptomatic infection was not yet available [15,16]. Several studies have quantified the difficulty of detecting cases by means of dynamic simulation modelling using empirical contact survey data [17,18]. Rapid detection has also been considered by applying new technology, such as the usage of digital contact tracing devices [19]. However, these studies have not systematically considered the feasibility of containment as the key outcome, and epidemiological parameters have been updated during the course of the ongoing pandemic.

The present study additionally investigates this matter, aiming to examine the feasibility of containment by identifying key factors that determines success within a simple stochastic model. Employing up-to-date parameter values for the natural history that complicates the control of COVID-19 infections, we apply a multi-type stochastic branching process model which allows to quantify the probability of extinction, following an introduction of imported cases. Accounting for the contribution of asymptomatic and pre-symptomatic individuals to secondary transmissions, the present study assesses the probability of a major epidemic given the number of untraced cases found in a location previously free of COVID-19.

## 2. Methods

A two-type branching process model was used to assess the probability of a major epidemic, assuming that a number of initial cases are untraced. To account for the asymptomatically infected individuals, we classified the type of host into symptomatic and asymptomatic. First, the next generation matrix of symptomatic and asymptomatic individuals can be formulated as

(1) $$K=\left(\begin{array}{cc} pR & qpR\\ \left(1-p\right)R & q\left(1-p\right)R \end{array}\right),$$

where *p* is the fraction of symptomatic infection (i.e., (1−p) is equal to the so-called “asymptomatic ratio”), q is the relative infectiousness among asymptomatically infected individuals compared to symptomatic individuals, and R is the value of reproduction number (the average number of secondary cases caused by a single symptomatic infected case in a fully susceptible population) among symptomatic cases.

Parameter values are partly empirically known. The proportion of symptomatic individuals (*p*) was set to be 0.6 as a baseline, because the asymptomatic ratio has been empirically estimated to range from 30 to 40%, for example, among passengers on the evacuation flights from Wuhan to Japan [4]. As part of the sensitivity analysis, we varied the value of *p* as 0.3 and 0.9, accounting for the possibility that the asymptomatic ratio ranges from 10% to 70% (Supplementary Figures S1–S3). The value of q, relative infectiousness, has not been explicitly quantified, and here we varied this value from 0.25 to 0.90, considering that the transmissibility (viral shedding) among asymptomatic individuals may be slightly lower than symptomatic cases and the absence of symptoms, such as coughing and sneezing, could reduce the droplets emitted. The basic reproduction number was varied from 1.5 to 3.5, given the wide range of estimates due to an ongoing pandemic [20].

In addition to the next-generation matrix (1), contact tracing and isolation can be implemented, and they can influence secondary transmissions from symptomatic individuals. Here, we pessimistically assume that the contract tracing reduces secondary transmission only among symptomatic cases: in reality, a part of transmissions from asymptomatic individuals may also be reduced, and thus, our exercise is regarded as yielding pessimistic scenarios. For simplicity, we ignore detection of asymptomatically infected individuals via active PCR testing. The next-generation matrix with interventions is rewritten as

(2) $$K^{\prime }=\left(\begin{array}{cc} \left(1-s\right)R+\alpha spR & qpR\\ \left(1-s\right)R+\alpha s\left(1-p\right)R & q\left(1-p\right)R \end{array}\right),$$

where *α* is the success rate of contacts traced and isolated, measured as the relative reduction in secondary transmissions only among symptomatic cases. Since approximately 40% (i.e., (1−s)=0.4) of transmission occurs prior to the symptom of onset, the relative reduction in secondary transmission due to successful contact tracing and isolation may only affect approximately 60% (i.e., s=0.6) of the secondary transmission from symptomatic individuals [3,21,22]. When interventions were in place, we assumed that all traced contacts were isolated.

Subsequently, the effective reproduction number under the intervention is calculated as

(3) $$R_{i}=\rho \left(K^{\prime }\right),$$

where ρ(·) stands for the largest eigenvalue. The probability of extinction π given a single infected case can be modeled as a multitype branching process of symptomatic and asymptomatic individuals as a probability-generating function of a negative binomial distribution [23]. The negative binomial distribution allows to incorporate the individual variation in infectiousness, varying the parameter k. Imposing an assumption for the success rate of contract tracing, finding the nonnegative root of the two quadratic equations with two unknown parameters, we were able to obtain the type-specific extinction probability from:

(4) $$\pi_{1}={\left(1+\frac{\left(1-s\right)+\alpha sp}{k}R\left(1-\pi_{1}\right)\right)}^{-k}{\left(1+\frac{\left(1-s\right)+\alpha s\left(1-p\right)}{k}R\left(1-\pi_{2}\right)\right)}^{-k},$$

(5) $$\pi_{2}={\left(1+\frac{qp}{k}R\left(1-\pi_{1}\right)\right)}^{-k}{\left(1+\frac{q\left(1-p\right)}{k}R\left(1-\pi_{2}\right)\right)}^{-k},$$

where *π*_1_ is the extinction probability given that a single symptomatic case is introduced, while *π*_2_ is the extinction probability given that an asymptomatically infected individual invades the new location. Throughout the main text, we assumed the overdispersion parameter of the offspring distribution k=1 (geometric distribution) as a baseline for the comparison of the type-specific heterogeneity (e.g., [24]), and the possibility of superspreading (individual variation in the number of secondary cases produced) was also considered in the Supplementary Figure S4 [25,26,27].

Here, we define the major epidemic as the chains of transmission that took off from stochastic fluctuations. In other words, the major epidemic is regarded as the large epidemic that does not probabilistically decline to extinction without extensive interventions [28]. One minus the probability of a major epidemic is equal to the probability of extinction. Taking the product of the two with the weight of the number of cases, the probability of a major epidemic is calculated as

(6) $$\mathrm{Pr}\left(major\;epidemic\right)=1-\pi_{1}^{\alpha pN}\pi_{2}^{(1-p)N},$$

where *N* is the total number of initially introduced cases, including asymptomatic infections. Of these, *pN* is symptomatic and (1 − *p*)*N* is asymptomatic. Out of *pN* symptomatic cases, *αpN* cases remain untraced. We considered the initial number of *N* to vary from 1, 5, 10, 15 to 20, and computed the probability of a major epidemic.

## 3. Results

**Figure 1:**
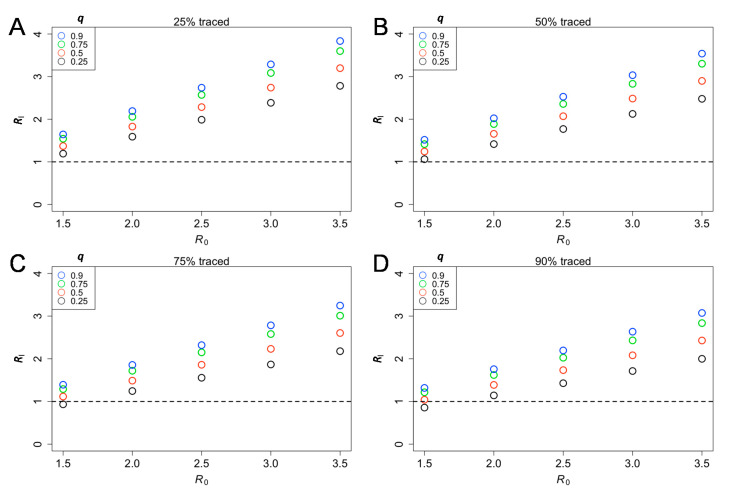
The effective reproduction number, given different effectiveness of contact tracing among symptomatic cases $\left(R_{i}\right)$ and the basic reproduction number ($R_{0}$). The probability of a major epidemic was estimated with different effectiveness levels of contact tracing (25% (α=0.75), 50% (α=0.5), 75% (α=0.25) and 90% (α=0.1) for panels **A**–**D**) among symptomatic cases. Colored circles classify the relative infectiousness of asymptomatic individuals compared with symptomatic individuals, varied from 0.25−0.90. The asymptomatic ratio was assumed at 40% (i.e., *p* = 0.6). Equation (3) was used to compute the eigenvalue.

Figure 1 shows the effective reproduction number $R_{i}$ varied by the proportion of symptomatic cases traced (Equation (3)). Overall, for *R_i_* to take the value below 1, a greater proportion of contacts must be traced as *R*_0_ increases. Assuming that the proportion of asymptomatic individuals was 40% as a baseline with 25% reduction in transmission among asymptomatic cases (*q* = 0.75), the probability of extinction given a symptomatic or asymptomatic individual with *α* = 0.25 (i.e., 75% of secondary transmissions were prevented by contact tracing and case isolation) and the reproduction number *R* among symptomatic cases at 2.5 was 32.0% and 38.8%, respectively (Equations (4) and (5)). Under these assumptions, even with 90% of symptomatic cases traced and isolated, the $R_{i}$ is not below the value of 1 (*R_i_* = 2.02), indicating the difficulty of containment when asymptomatic individuals persist in their capacity to transmit to susceptible individuals (Figure 1D).

**Figure 2:**
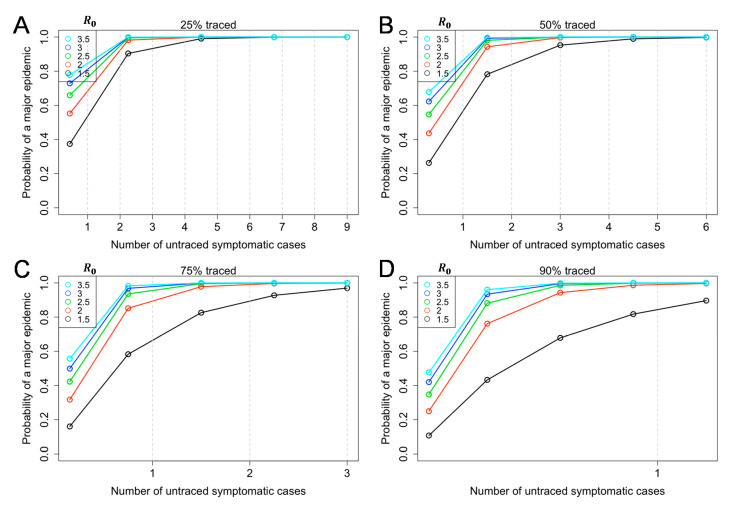
Probability of a major epidemic, given the number of untraced symptomatic cases and the reproduction number, *R*. Equation (6) in the main text was used. The probability of a major epidemic was estimated given different levels of success in contact tracing (25% (α=0.75), 50% (α=0.5), 75% (α=0.25), and 90% (α=0.1) for panels **A**–**D**) among symptomatic cases. Colored lines classify the reproduction number among symptomatic cases varied from 1.5–3.5. The relative infectiousness among asymptomatic individuals was assumed to be 75% (*q* = 0.75). The asymptomatic ratio was assumed as 40% (i.e., *p* = 0.6). The number of untraced symptomatic cases is below 1 in Figure 2D.

Figure 2 shows the probability of a major epidemic given by the number of untraced symptomatic cases and the reproduction number, *R* among symptomatic cases (Equation (6)). If the success rate of contacts traced and isolated (α) is high, the probability of a major epidemic may be high even by the time of observing one untraced case. Even if the reproduction number is 1.5, and even if 90% of symptomatic individuals are traced and isolated, the probability of a major epidemic exceeds 80% (Figure 1D).

In reality, the $R_{i}$ may decrease after generations, since awareness of the disease will encourage non-pharmaceutical interventions to be implemented (e.g., wearing masks and washing hands) [29]. Sometimes, unnecessary risky contact may be avoided upon recognition of the epidemic. Such an impact has been empirically observed historically (e.g., [30])). To incorporate such a temporal effect of social behavior, one can formulate the time-varying reproduction number R(t) as,

(7) $$R\left(t\right)=R_{i}\exp\left(-\delta t\right),$$

where δ is the rate of decay due to increased awareness. While such an effect cannot be completely ignored, empirically quantifying the population effect of social behavior at the beginning of disease introduction has not been capable in any location, and thus is not considered in the present analysis. Thus, we note that in the present study, $R_{i}$ may be overestimated compared to the reality.

**Figure 3:**
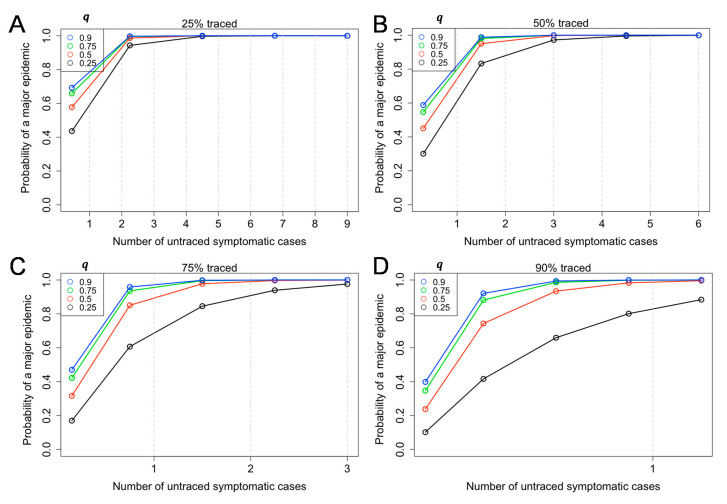
Probability of a major epidemic, given the number of untraced symptomatic cases and the relative infectiousness of an asymptomatic individual (q). Equation (6) in the main text was used. The probability of a major epidemic was estimated given different rates of success in contact tracing (25% (α=0.75), 50% (α=0.5), 75% (α=0.25), and 90% (α=0.1) for panels **A**–**D**) among symptomatic cases. Colored circles and lines classify the relative infectiousness of asymptomatic individuals compared to symptomatic individuals (q), which was assumed to vary from 0.25–0.90. The reproduction number among symptomatic cases was assumed as *R* = 2.5. The asymptomatic ratio was assumed as 40% (i.e., *p* = 0.6). The number of untraced symptomatic cases is below 1 in Figure 3D.

Figure 3 shows the probability of a major epidemic given by the number of untraced symptomatic cases and the relative transmissibility among asymptomatic individuals (q) (Equation (6)). Overall, the probability of a major outbreak will increase with the number of untraced symptomatic cases introduced to the population (Figure 3). If *q* is as high as 90% (i.e., *q* = 0.9), the containment of COVID-19 would be very challenging, with the baseline assumption that 40% are asymptomatic and *R* = 2.5 (Figure 3A–D). For instance, regardless of the relative infectiousness of asymptomatic individuals, provided that 75% of symptomatic individuals were traced and isolated, with the presence of three untraced symptomatic cases, the probability of a major epidemic exceeds 97% for all values of *q* (Figure 3C).

## 4. Discussion

The present study examined the probability of a major epidemic given by a certain number of untraced symptomatic cases. We were motivated to conduct this experiment because only the number of traced symptomatic cases are recorded in a timely manner, while other aspects, especially asymptomatic infections, are unobserved. As the value of *R_i_* was above the value of one (Figure 1), the probability of a major outbreak will increase with the number of untraced symptomatic cases introduced to the population (Figure 2 and Figure 3). A broad range of asymptomatic ratios and relative infectiousness among asymptomatic cases was assumed, and they were shown to complicate the containment strategies of COVID-19. Moreover, since approximately 40% of secondary transmissions can occur prior to symptom onset, contact tracing and case isolating only after illness onset is difficult to prevent the epidemic to take off. Thus, our result emphasizes the value of active contact tracing among close contacts of an infected individual to identify and isolate asymptomatic and pre-symptomatic cases. Considering that the probability of successful containment by contact tracing and case isolation is determined by detailed ingredients of the next generation matrix, and thus basic reproduction number *R*, updating the estimated value of the *R* in real time is thus considered to be critical to judge the feasibility of containment in a timely manner.

While achieving 90% contact tracing and isolation among symptomatic cases may be impossible, there can be a scenario in which the probability of a major epidemic is very high, even when there is only a single untraced case (Figure 3D). That is, there can be a substantial number of asymptomatically infected individuals who are fully untraced, with comparable size to the total volume of symptomatic cases, and they may possess a comparable transmissibility to symptomatic cases. We have shown that the transmissibility among asymptomatic individuals would be a key parameter to determine whether containment is feasible. Even if isolation of symptomatic cases is conducted swiftly after the symptom onset, only secondary transmissions after the symptom onset can be prevented, and also, the presence asymptomatic cases continues to maintain the value of $R_{i}$ high enough for the epidemic to take off. In addition to published studies on contact tracing of COVID-19 with and without digital tracing applications [15,16,17,18,19], the present study is the first to have explicitly modeled the probability of extinction with an introduction of a single symptomatic or asymptomatic case.

Because controlling the disease by case-isolating focuses on symptomatic confirmed cases, asymptomatic cases are fully uncontrolled without quarantine measures. Our analysis highlighted the importance of identifying potential asymptomatic cases by means of active contact tracing. By rapidly identifying and isolating the asymptomatic contacts, the combination measures will help to decrease the probability of a major epidemic. Thus, developing a rapid diagnostic testing kit for COVID-19 is deemed important to ensure that cases can be isolated before producing additional generations of cases.

Several limitations of this study must be noted. First, while the model scenarios relied on how well symptomatic cases are traced, actual validation of this proportion in real time was not feasible, as the ascertainment rate may be quite low during an exponential growth phase of the epidemic [31]. Second, the model assumed that when contact tracing is achieved, this case will not produce any secondary cases, while in reality, secondary transmission may occur before case identification and during the process of isolation. Moreover, the model could not account for possible reduction in asymptomatic transmissions due to contact tracing, while we have successfully quantified the importance to enhance timely active surveillance to identify asymptomatic and pre-symptomatic cases. Third, in the case where a location has the capacity to conduct active surveillance of close contacts of an infected individual, isolation of asymptomatic cases may be achieved, which would contribute to the extinction of the disease. However, in our present analysis, we did not consider the effect of isolating asymptomatic individuals, as we focused on the number of untraced symptomatic cases because we believed it would complicate the interpretation.

Despite the number of these simplicities, we believe that our model still captures the essence to describe the difficulty of containment of COVID-19. In conclusion, containment of COVID-19 is more challenging than SARS and MERS. Collating published evidence of the natural history of COVID-19 to the present day, a simple model can help quantify the probability of a major epidemic. As the new technology is developed to rapidly identify both symptomatic and asymptomatic cases, the feasibility of containment may be improved with active surveillance in the future.

## Supplementary Materials

The following are available online at https://www.mdpi.com/2077-0383/9/10/3125/s1, Figure S1: Sensitivity analysis of Figure 1, considering the asymptomatic rate of 70%, and 10% (i.e., *p* = 0.3 and *p* = 0.9) and different effectiveness of success in contact tracing (25% (*α* = 0.75), 50% (*α* = 0.5), 75% (*α* = 0.25) and 90% (*α* = 0.1)), Figure S2: Sensitivity analysis of Figure 2, considering the asymptomatic rate of 70%, and 10% (i.e., *p* = 0.3 and *p* = 0.9) and different effectiveness of success in contact tracing (25% (*α* = 0.75), 50% (*α* = 0.5), 75% (*α* = 0.25) and 90% (*α* = 0.1)), Figure S3: Sensitivity analysis of Figure 3, considering the asymptomatic rate of 70%, and 10% (i.e., *p* = 0.3 and *p* = 0.9) and different effectiveness of success in contact tracing (25% (*α* = 0.75), 50% (*α* = 0.5), 75% (*α* = 0.25) and 90% (*α* = 0.1)), Figure S4: Probability of a major epidemic using negative binomial distribution.
