# Supplementary material for: Containment, Contact Tracing and Asymptomatic Transmission of Novel Coronavirus Disease (COVID-19): A Modelling Study
Source: J Clin Med. 2020 Sep 27;9(10):3125. doi: 10.3390/jcm9103125 (PMC7600034; doi:10.3390/jcm9103125)
Supplement: Supplementary file 1 [file jcm-09-03125-s001.pdf]

Appendix. Supplementary Materials

(1) Sensitivity analysis of asymptomatic rate

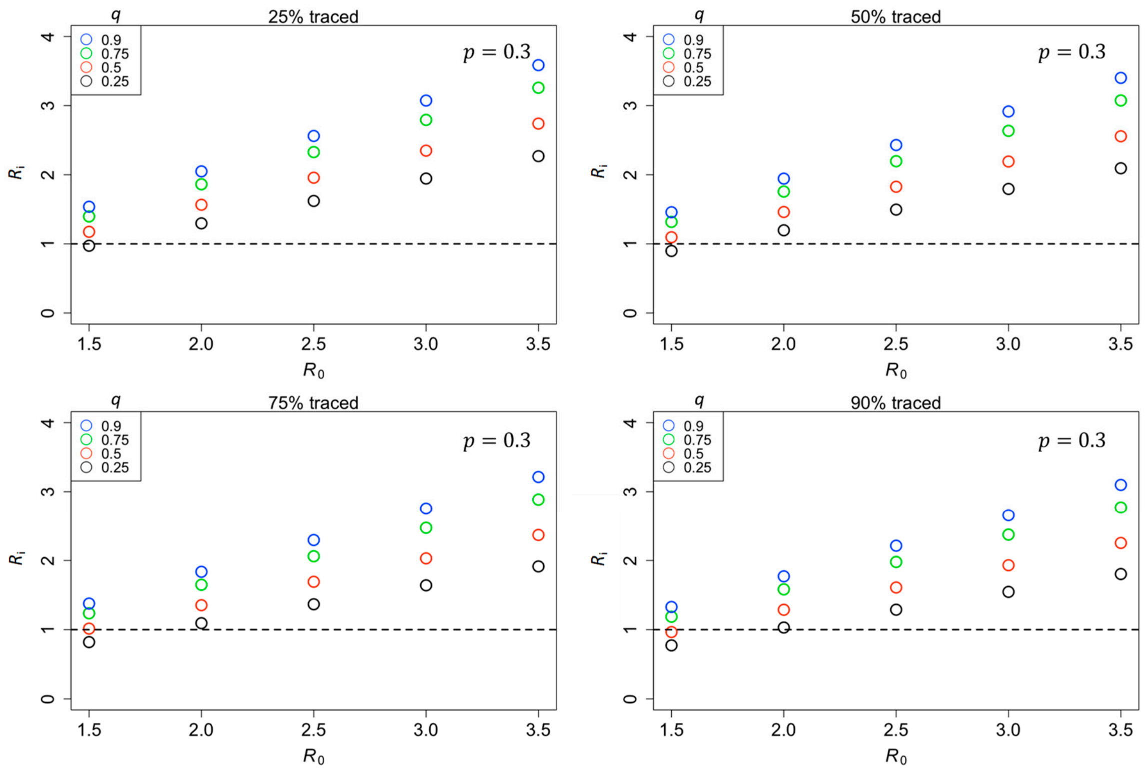

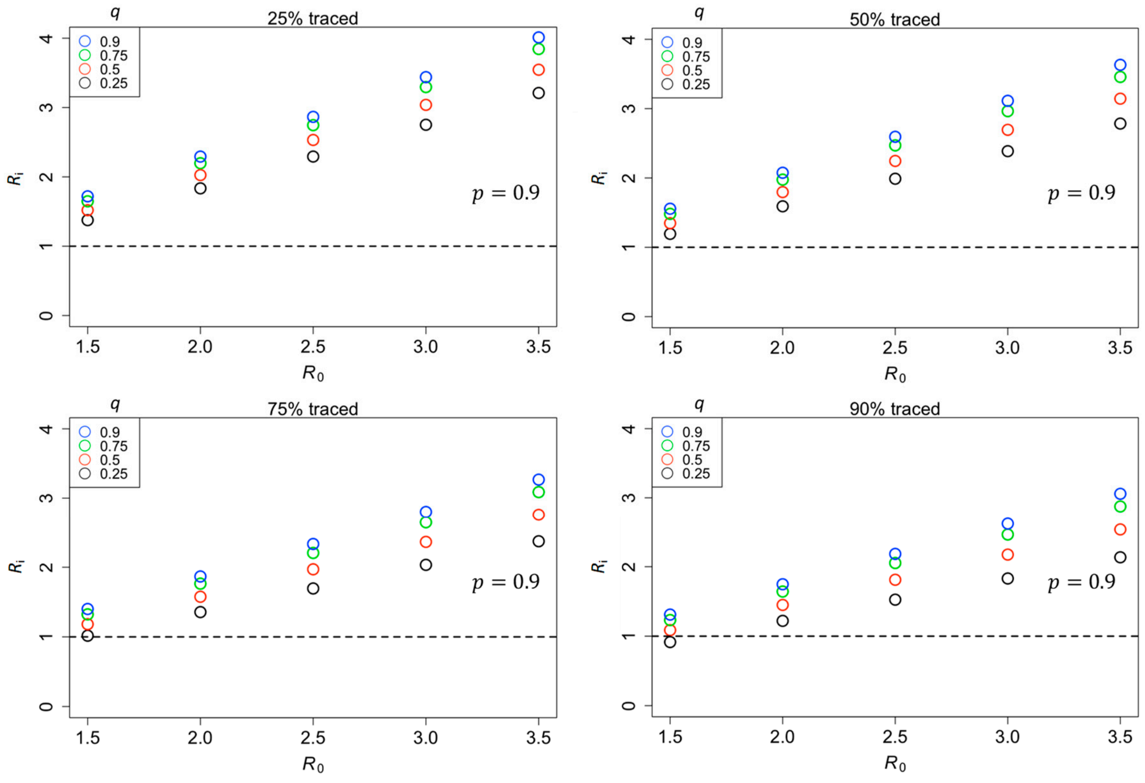

Supplementary Figure 1. Sensitivity analysis of Figure 1, considering the asymptomatic rate of 70%, and 10% (i.e., p=0.3 and p=0.9) and different effectiveness of success in contact tracing (25% ($\alpha = 0.75$), 50% ($\alpha = 0.5$), 75% ($\alpha = 0.25$) and 90% ($\alpha = 0.1$)).

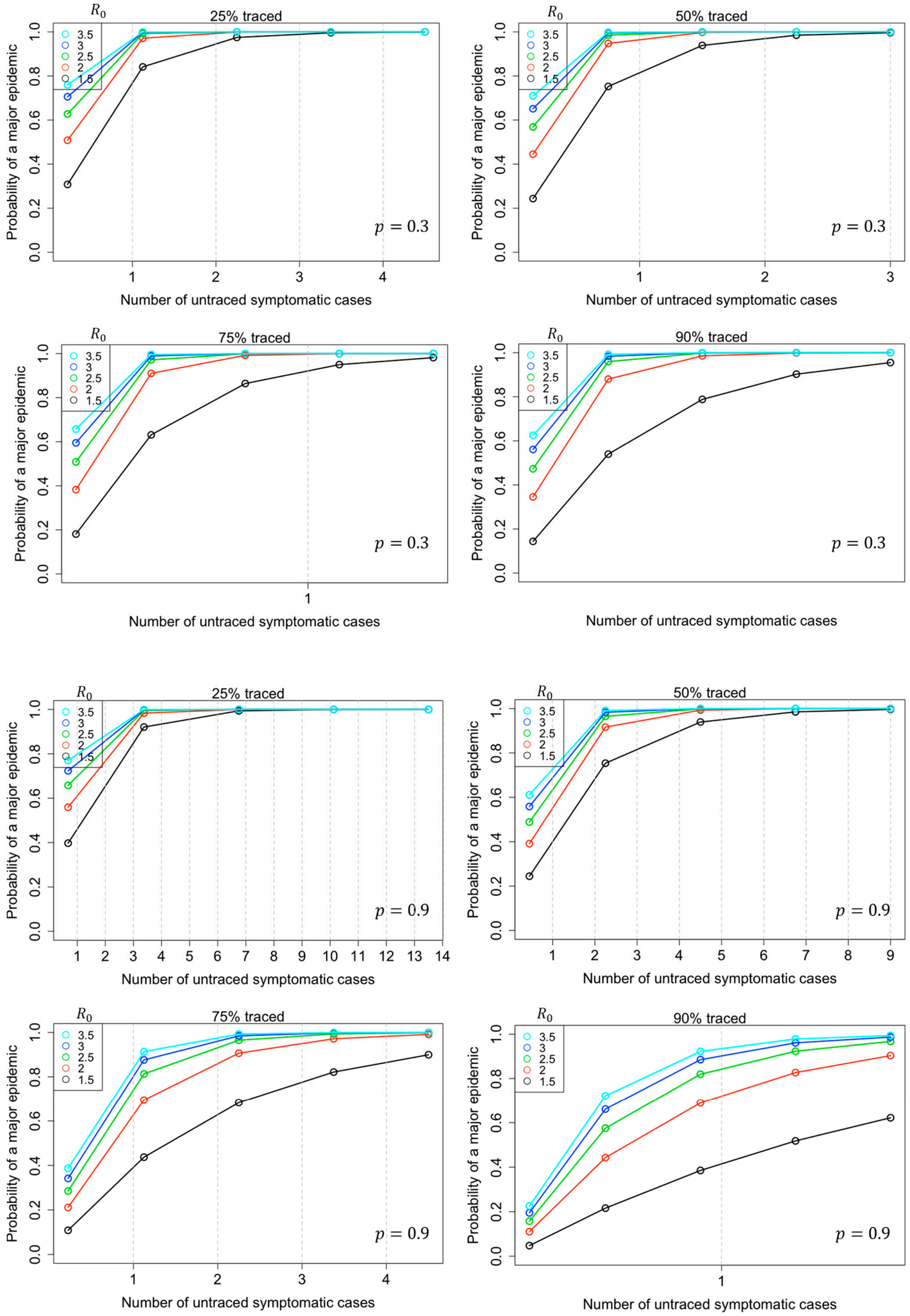

Supplementary Figure 2. Sensitivity analysis of Figure 2, considering the asymptomatic rate of 70%, and 10% (i.e., p=0.3 and p=0.9) and different effectiveness of success in contact tracing (25% ($\alpha = 0.75$), 50% ($\alpha = 0.5$), 75% ($\alpha = 0.25$) and 90% ($\alpha = 0.1$)).

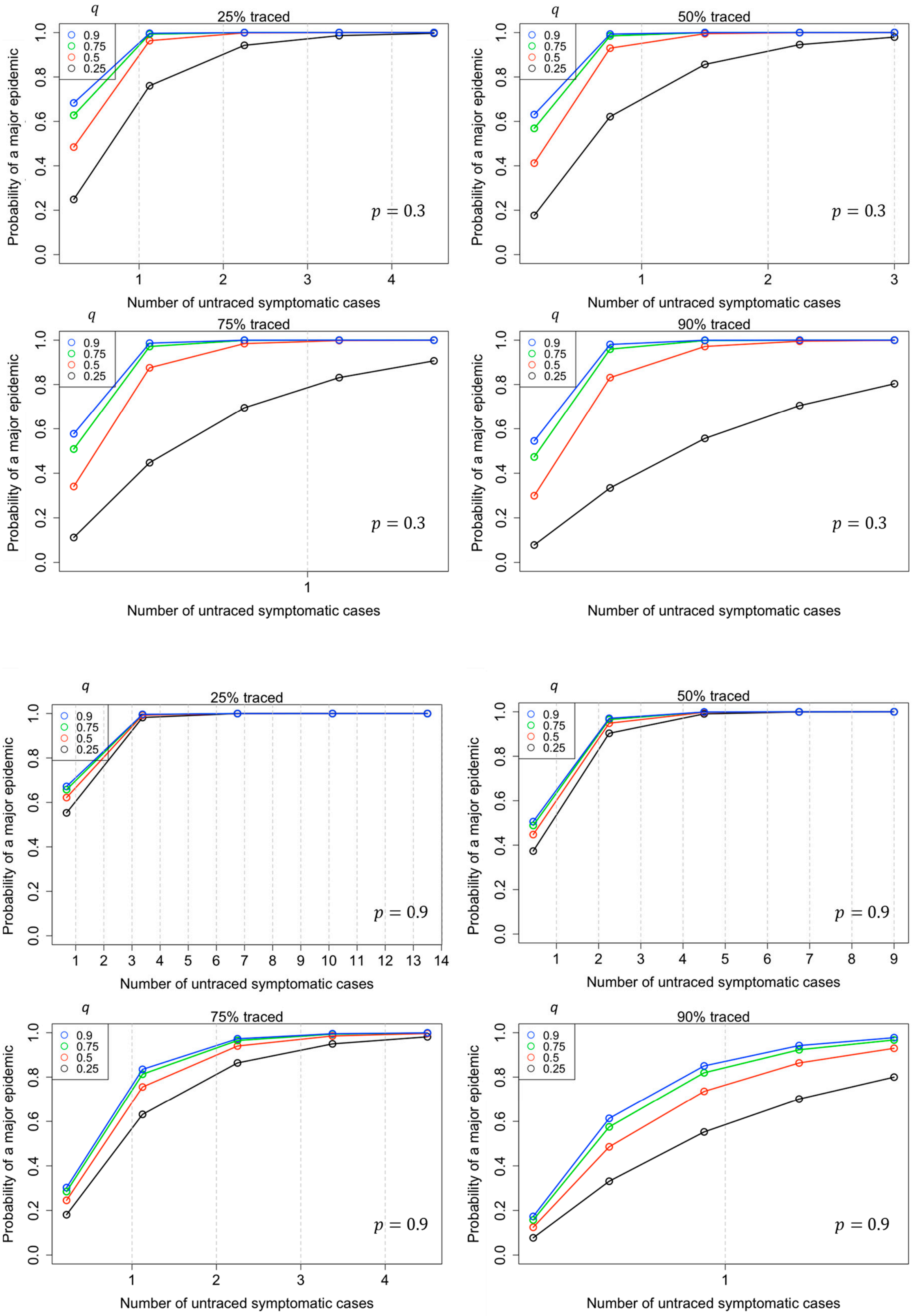

Supplementary Figure 3. Sensitivity analysis of Figure 3, considering the asymptomatic rate of 70%, and 10% (i.e., p=0.3 and p=0.9) and different effectiveness of success in contact tracing (25% ($\alpha = 0.75$), 50% ($\alpha = 0.5$), 75% ($\alpha = 0.25$) and 90% ($\alpha = 0.1$)).

(2) Accounting for superspreading using negative binomial distribution

The dispersion parameter k for COVID-19 has been estimated to be around 0.1 suggesting that 80% of secondary cases may have been caused by a small fraction of infected individuals (superspreading events) [13]. Varying the parameter k and applying Equation (6), the probability of a major epidemic can be calculated (Supplementary Figure 4).

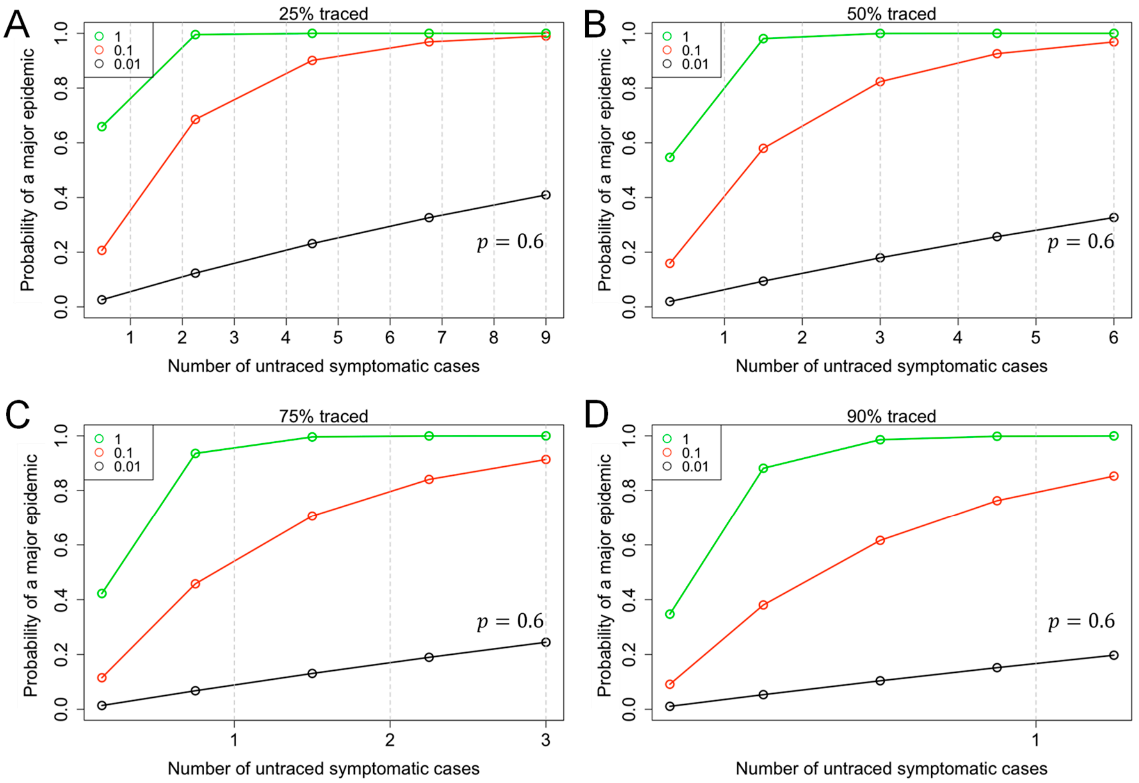

Supplementary Figure 4. Probability of a major epidemic using negative binomial distribution. The dispersion parameter (k) was varied from 0.01 (overdispersed) to 1 (geometric). The probability of a major epidemic was estimated given different rates of success in contact tracing (25% (α = 0.75), 50% (α = 0.5), 75% (α = 0.25) and 90% (α = 0.1) for panels A, B, C and D) among symptomatic cases. The reproduction number among symptomatic cases was assumed as R = 2.5. The asymptomatic ratio was assumed as 40% (i.e., p = 0.6).
